# The impact of marital status on gastric cancer-related prognosis: A propensity-score matched study

**DOI:** 10.1097/MD.0000000000050761

**Published:** 2026-09-18

**Authors:** Haitao Ma, Qiang Bing, Xuhao Duan, Genwu Yao, Bichen Wang, Yaozu Bao, Yongyong Liu

**Affiliations:** a Department of General Surgery, First People’s Hospital of Lanzhou, Lanzhou, Gansu, China.

**Keywords:** Cox regression, gastric cancer, marital status, propensity-score matching, survival disparity

## Abstract

Gastric cancer remains a leading gastrointestinal malignancy with persistently high incidence and poor prognosis. Marital status, an established proxy for social support, has been linked to survival in several solid tumors, yet its impact on gastric-cancer outcomes remains uncertain in large contemporary cohorts. The aim of this study was to quantify the association between marital status and cancer-specific and overall survival in patients with gastric cancer in a large, contemporary, population-based cohort, and to determine whether marital status serves as an an independent prognostic factor for gastric-cancer outcomes. Using the surveillance, epidemiology, and end results database, we identified patients with primary gastric adenocarcinoma diagnosed from 2013 through 2022. Patients were dichotomized as married or unmarried. Baseline characteristics were compared with χ^2^ tests. To mitigate selection bias, 1:1 propensity-score matching (PSM) was performed. Cancer-specific survival (CSS) and overall survival (OS) were estimated with Kaplan–Meier and log-rank tests. Subgroup analyses and multivariable Cox regression were conducted to determine the independent prognostic value of marital status. A total of 39,013 patients were included (median follow-up 14 months). Before PSM, unmarried patients had significantly worse CSS (hazard ratio [HR] 1.17, 95% confidence interval [CI] 1.14–1.20, *P* < .001) and OS (HR 1.20, 95% CI 1.16–1.23, *P* < .001). After 1:1 PSM (15,256/group), the survival gap narrowed but remained significant (CSS: HR 1.12, *P* < .001; OS: HR 1.15, *P* < .001). Subgroup analyses showed unmarried status was consistently associated with poorer CSS and OS across most strata. Multivariable Cox regression confirmed unmarried status as an independent adverse prognostic factor for both CSS (HR 1.17, 95% CI 1.13–1.20, *P* < .001) and OS (HR 1.19, 95% CI 1.15–1.22, *P* < .001). In this large population-based cohort, unmarried status was independently associated with worse gastric-cancer outcomes. Marital status is a readily available marker of social and economic support that identifies patients at higher risk of adverse outcomes; these patients may benefit from targeted psychosocial, financial, and logistical support during and after treatment.

## 1. Introduction

Gastric cancer is one of the most common gastrointestinal malignancies worldwide; its incidence and mortality have remained persistently high, constituting a major threat to public health.^[1]^ Although diagnostic and therapeutic advances over the past decade have been realized, long-term survival is still disappointing – particularly for patients who present with advanced disease.^[2]^ Gastric carcinogenesis is a multifactorial, multistep process driven by complex interactions among environmental exposures, genetic susceptibility, and lifestyle factors.^[3]^ Beyond tumor biology itself, sociodemographic characteristics can materially influence disease trajectory and prognosis. Among these, marital status – an easily ascertained proxy for social support – has emerged as a potentially modifiable determinant of cancer outcomes.

The interplay between marital status and health endpoints has been examined across numerous chronic diseases and solid tumors. A systematic review and meta-analysis encompassing >6 million cancer patients demonstrated that unmarried status is associated with significantly higher cancer-specific and overall mortality.^[4]^ In a landmark analysis of the 10 leading causes of cancer death in the United States, Aizer et al showed that married patients were less likely to present with metastatic disease, more likely to receive definitive therapy, and less likely to die of cancer than their unmarried counterparts.^[5]^ Marriage is generally associated with healthier behaviors, superior treatment adherence, and more robust psychosocial support, all of which may enhance disease management and extend survival.^[6]^ Data specific to gastric cancer have also accumulated but remain inconsistent. Using earlier releases of the surveillance, epidemiology, and end results (SEER) database, Jin et al and Zhou et al reported that married patients with gastric cancer had significantly better survival than unmarried patients;^[7,8]^ Li and Hu further demonstrated that the protective effect of marriage varies with time since diagnosis^[9]^; and studies restricted to gastric adenocarcinoma and gastric neuroendocrine neoplasms reached broadly similar conclusions.^[10,11]^ However, these studies were based on data collected largely before 2012, were limited by modest sample sizes, or were restricted to single histologic subtypes, leaving their conclusions vulnerable to temporal and selection bias. As staging practice and multimodal therapy have evolved over the past decade, whether the survival advantage of married patients persists in the contemporary treatment era remains uncertain. Clarifying this association is clinically important: if marital status independently modulates outcome, it could be incorporated into risk-stratified management, and patients lacking spousal support could be targeted for intensified psychosocial and logistical support.

Accordingly, we leveraged the most recent decade of data from the SEER program – the largest population-based cancer registry globally – to quantify survival differences between married and unmarried patients with gastric cancer. We hypothesized that unmarried status would be associated with worse cancer-specific and overall survival, and we sought to provide a robust, contemporary benchmark for future translational and intervention studies.

## 2. Patients and methods

### 2.1. Data source and data collection

The SEER program, maintained by the U.S. National Cancer Institute, is a network of population-based cancer registries that prospectively collects cancer incidence, treatment, and survival data from geographically diverse U.S. populations. Within each participating registry, trained cancer registrars abstract demographic, tumor, treatment, and follow-up information from hospital and outpatient medical records, pathology and laboratory reports, radiotherapy and oncology facilities, and death certificates, following standardized data-collection and coding protocols established by the U.S. National Cancer Institute and the North American Association of Central Cancer Registries, with routine data-quality audits. The current study used the SEER-17 registries database (November 2024 submission), which covers approximately 26.5% of the total U.S. population. Data were extracted by the authors on October 20, 2025 using SEERStat software, version 9.0.42 (Surveillance Research Program, National Cancer Institute, Bethesda). For each eligible patient, we collected demographic variables (year of diagnosis, age at diagnosis, sex, race, ethnicity, marital status at diagnosis, county-level median household income, and urban/rural residence), tumor characteristics (International Classification of Diseases for Oncology, 3rd edition topography codes C16.0–C16.9, histologic type, grade, and SEER summary stage), 1st-course treatment modalities (surgery, radiotherapy, and chemotherapy), and outcome variables (vital status, SEER cause-specific death classification, and survival time in months). The use of such data for research purposes is generally exempt from ethical review according to the “Ethical Review Methods for Health-Related Research Involving Human Participants” as outlined by the World Health Organization, which states that research using publicly available, de-identified data does not require ethical review if it does not involve any form of interaction or intervention with individual participants. Therefore, ethical approval and informed consent were waived.

### 2.2. Patient selection

**Figure 1.**
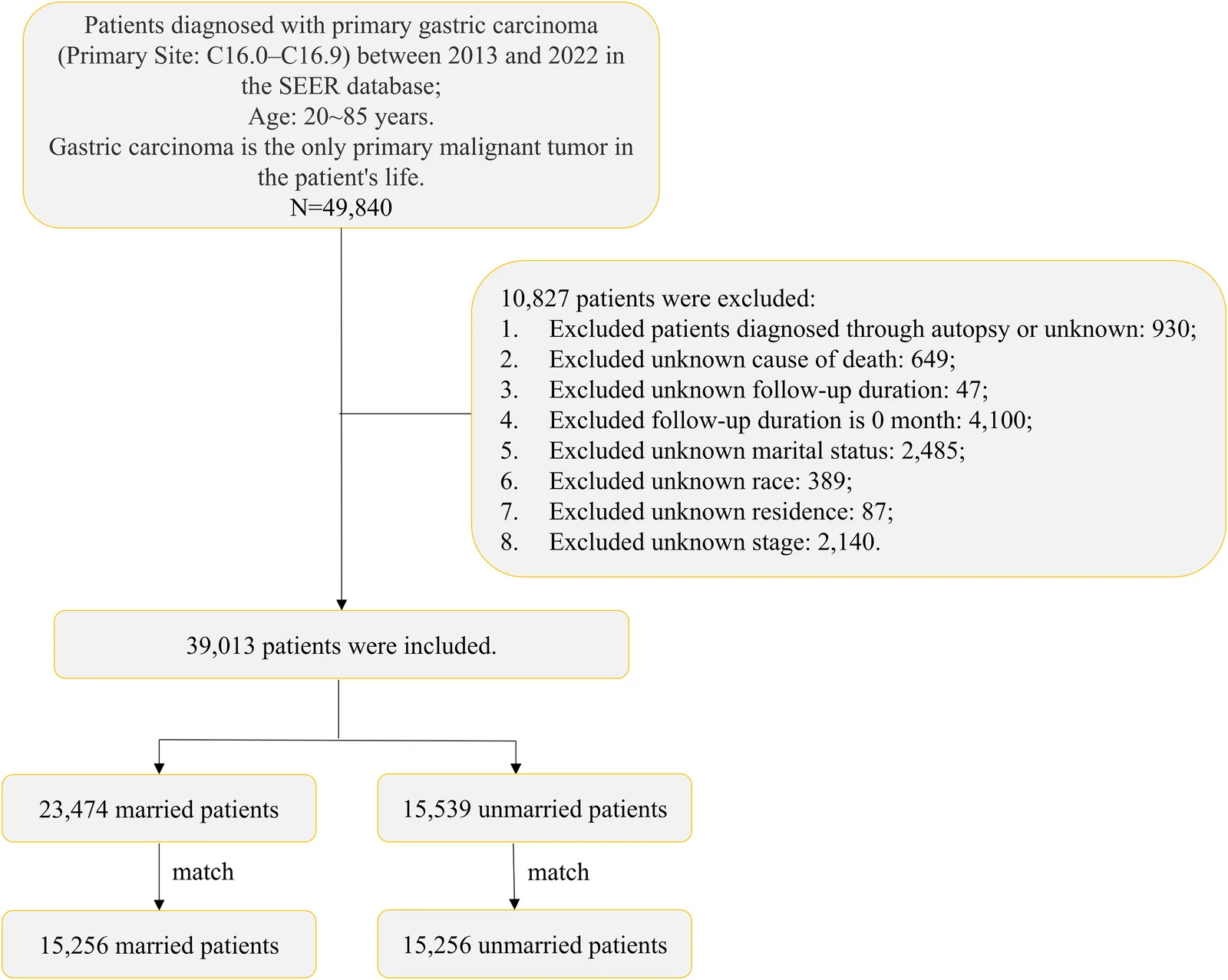
Patient screening flowchart. SEER = surveillance, epidemiology, and end results.

From SEER-17 we selected patients aged 20 to 85 years who received a diagnosis of primary gastric solid tumor (International Classification of Diseases for Oncology, 3rd edition topography C16.0–C16.9) during 2013 to 2022 and for whom gastric cancer was the only lifetime primary malignancy. We excluded patients whose tumor was detected at autopsy or whose diagnostic method was unknown, patients whose cause of death was unknown, and patients whose follow-up time was unknown or equal to 0. In addition, patients whose marital status, race, place of residence, or stage was unknown were not included in the final analysis. The patient-selection flowchart is shown in Figure 1. A total of 39,013 patients were finally enrolled.

### 2.3. Variable processing

Using the SEER coding system, we obtained demographic characteristics (year of diagnosis, age, marital status, sex, race, ethnicity, median household income, and place of residence), tumor characteristics (histologic type, grade, stage), and treatment modalities (surgery, radiotherapy, and chemotherapy). Patient outcome (cancer-specific survival [CSS] and overall survival [OS]) was determined from vital status and follow-up time. In the 2000 to 2022 SEER-17 dataset, median household income (inflation-adjusted to 2023) was categorized as <40,000, 40,000 to 44,999, 45,000 to 49,999, 50,000 to 54,999, 55,000 to 59,999, 60,000 to 64,999, 65,000 to 69,999, 70,000 to 74,999, 75,000 to 79,999, 80,000 to 84,999, 85,000 to 89,999, 90,000 to 94,999, 95,000 to 99,999, 100,000 to 109,999, 110,000 to 119,999 and ≥120,000 USD. Marital status was recorded as married, single (never married), separated, divorced, widowed, and unmarried or domestic partner.

For subsequent analyses, we grouped year of diagnosis into 2013 to 2017 and 2018 to 2022, age at diagnosis into 20 to 49, 50 to 64, 65 to 74 and 75 to 85 years; marital status into married and unmarried (single, separated, divorced, widowed, unmarried, or domestic partner); sex into male and female; race into White, Black, and other (Asian or Pacific Islander and American Indian/Alaska Native); ethnicity into Hispanic and non-Hispanic; median household income into <75,000 and ≥75,000 USD; and place of residence into urban and rural.

### 2.4. Statistical analysis

Patients were divided into married and unmarried groups. Between-group differences in baseline characteristics were evaluated with Pearson χ^2^ tests. The outcomes of interest were CSS and OS. Kaplan–Meier curves and the log-rank test were used to compare CSS and OS between the 2 groups. To reduce the influence of baseline imbalance on survival, we then performed 1:1 propensity-score matching (PSM). After PSM, Kaplan–Meier curves and log-rank tests were reapplied to assess the effect of marital status on CSS and OS. In addition, subgroup analyses based on prespecified variables were carried out, and multivariable Cox regression was employed to verify the independent influence of marital status on CSS and OS. Finally, to assess heterogeneity within the unmarried group, we additionally performed a sensitivity analysis in which marital status was modeled as a 6-level variable (married [reference], single [never married], separated, divorced, widowed, and unmarried or domestic partner), using Kaplan–Meier analysis and multivariable Cox regression with the same covariate adjustment as the primary analysis. All statistical analyses were performed with R software, version 4.3.0 (R Foundation for Statistical Computing, Vienna, Austria). All *P* values were 2-sided, and the significance threshold was set at .05.

To assess the robustness of the findings to unmeasured confounding, *E* values were computed for the main multivariable hazard ratios (HRs); the *E* value quantifies the minimum strength of association that an unmeasured confounder would need to have with both marital status and survival, above and beyond the adjusted covariates, to fully explain away the observed association.^[12]^

### 2.5. Follow-up and outcomes

SEER registries conduct annual active and passive follow-up of all registered patients. Vital status is ascertained through hospital and physician contact, state vital statistics offices, and linkage with the national death index; follow-up for the 2000 to 2022 SEER-17 dataset is complete through December 31, 2022. CSS was defined as the interval from diagnosis to death attributed to gastric cancer according to the SEER cause-specific death classification, and OS as the interval from diagnosis to death from any cause. Patients alive at the last follow-up date were censored.

## 3. Results

### 3.1. Baseline patient characteristics

A total of 39,013 patients met the eligibility criteria and were included in the final analysis (Fig. 1). Table 1 summarizes the baseline characteristics of the 39,013 patients, who had a median follow-up of 14 months. Of these, 23,474 (60.17%) were married and 15,539 (39.83%) were unmarried. 17,602 patients (45.12%) were diagnosed in 2013 to 2017 and 21,411 (54.88%) in 2018 to 2022. The age distribution was as follows: 5720 (14.66%) were aged 20 to 49 years, 13,679 (35.07%) aged 50 to 64 years, 11,623 (29.79%) aged 65 to 74 years, and 7991 (20.48%) aged 75 to 85 years. 23,493 patients (60.22%) were male and 15,520 (39.78%) were female. Demographically, most patients were White (70.18%), non-Hispanic (76.69%), had a median household income ≥$75,000 (69.14%), and resided in urban areas (90.43%). Histologically, 23,043 tumors (59.06%) were adenocarcinomas, 5422 (13.90%) were signet-ring cell carcinomas, 2432 (6.23%) were neuroendocrine tumors, 4727 (12.12%) were gastrointestinal stromal sarcomas, and 3389 (8.69%) were other types. Grade distributions were 3164 (8.11%) grade I, 9013 (23.10%) grade II, 17,561 (45.02%) grade III, 379 (0.97%) grade IV, and 8896 (22.80%) unknown. Stage at diagnosis was localized in 13,436 patients (34.44%), regional in 10,525 (26.98%), and distant in 15,052 (38.58%). Treatment modalities included surgery in 18,630 (47.75%), radiotherapy in 8549 (21.91%), and chemotherapy in 23,368 (59.90%).

**Table 1:** Patient demographics and baseline characteristics.

Characteristics	Unmatched			Matched		
	Married N = 23,474*	Unmarried N = 15,539*	*P* value†	Married N = 15,256*	Unmarried N = 15,256*	SMD‡
Year of diagnosis			.031			
2013–2017	10,695 (46%)	6907 (44%)		6825 (45%)	6816 (45%)	−0.001
2018–2022	12,779 (54%)	8632 (56%)		8431 (55%)	8440 (55%)	0.001
Age			<.001			
20–49	3210 (14%)	2510 (16%)		2523 (17%)	2434 (16%)	−0.016
50–64	8311 (35%)	5368 (35%)		5592 (37%)	5259 (34%)	−0.046
65–74	7418 (32%)	4205 (27%)		4155 (27%)	4158 (27%)	0.000
75–85	4535 (19%)	3456 (22%)		2986 (20%)	3405 (22%)	0.066
Sex			<.001			
Male	15,581 (66%)	7912 (51%)		8085 (53%)	7905 (52%)	−0.024
Female	7893 (34%)	7627 (49%)		7171 (47%)	7351 (48%)	0.024
Race			<.001			
White	16,838 (72%)	10,542 (68%)		11,172 (73%)	10,533 (69%)	−0.090
Black	2229 (9%)	3097 (20%)		2225 (15%)	2823 (19%)	0.098
Other	4407 (19%)	1900 (12%)		1859 (12%)	1900 (12%)	0.008
Ethnicity			<.001			
Hispanic	5257 (22%)	3836 (25%)		3957 (26%)	3824 (25%)	−0.020
Non-Hispanic	18,217 (78%)	11,703 (75%)		11,299 (74%)	11,432 (75%)	0.020
Median household income			<.001			
<75,000 USD	6866 (29%)	5173 (33%)		4867 (32%)	5045 (33%)	0.025
≥75,000 USD	16,608 (71%)	10,366 (67%)		10,389 (68%)	10,211 (67%)	−0.025
Residence			.175			
Urban	21,267 (91%)	14,014 (90%)		13,792 (90%)	13,746 (90%)	−0.010
Rural	2207 (9%)	1525 (10%)		1464 (10%)	1510 (10%)	0.010
Histology			.002			
Adenocarcinoma	14,036 (60%)	9007 (58%)		8820 (58%)	8904 (58%)	0.011
Signet-ring cell carcinoma	3249 (14%)	2173 (14%)		2169 (14%)	2142 (14%)	−0.005
Neuroendocrine tumor	1438 (6%)	994 (6%)		1032 (7%)	972 (6%)	−0.016
Gastrointestinal stromal sarcoma	2793 (12%)	1934 (12%)		1900 (12%)	1832 (12%)	−0.014
Other	1958 (8%)	1431 (9%)		1335 (9%)	1406 (9%)	0.016
Grade			<.001			
Grade I	1891 (8%)	1273 (8%)		1317 (9%)	1253 (8%)	−0.015
Grade II	5465 (23%)	3548 (23%)		3436 (23%)	3451 (23%)	0.002
Grade III	10,748 (46%)	6813 (44%)		6846 (45%)	6722 (44%)	−0.016
Grade IV	224 (1%)	155 (1%)		130 (1%)	153 (1%)	0.015
Unknown	5146 (22%)	3750 (24%)		3527 (23%)	3677 (24%)	0.023
Stage			.042			
Localized	8000 (34%)	5436 (35%)		5360 (35%)	5283 (35%)	−0.011
Regional	6434 (27%)	4091 (26%)		3938 (26%)	4053 (27%)	0.017
Distant	9040 (39%)	6012 (39%)		5958 (39%)	5920 (39%)	−0.005
Local surgery			<.001			
Yes	11,642 (50%)	6988 (45%)		7010 (46%)	6831 (45%)	−0.024
No/unknown	11,832 (50%)	8551 (55%)		8246 (54%)	8425 (55%)	0.024
Radiotherapy			<.001			
Yes	5384 (23%)	3165 (20%)		3174 (21%)	3141 (21%)	−0.005
No/unknown	18,090 (77%)	12,374 (80%)		12,082 (79%)	12,115 (79%)	0.005
Chemotherapy			<.001			
Yes	14,720 (63%)	8648 (56%)		8751 (57%)	8521 (56%)	−0.030
No/unknown	8754 (37%)	6891 (44%)		6505 (43%)	6735 (44%)	0.030

* n (%).

† Pearson chi-squared test.

‡ Standardized mean difference.

Before PSM, married and unmarried patients differed significantly in year of diagnosis (*P* = .031), age (*P* < .001), sex (*P* < .001), race (*P* < .001), ethnicity (*P* < .001), median household income (*P* < .001), histologic type (*P* = .002), tumor grade (*P* < .001), stage (*P* = .042), surgery (*P* < .001), radiotherapy (*P* < .001), and chemotherapy (*P* < .001). After 1:1 PSM, 15,256 married and 15,256 unmarried patients were retained, with standardized mean differences <0.1 for all covariates.

### 3.2. Survival outcomes

**Figure 2.**
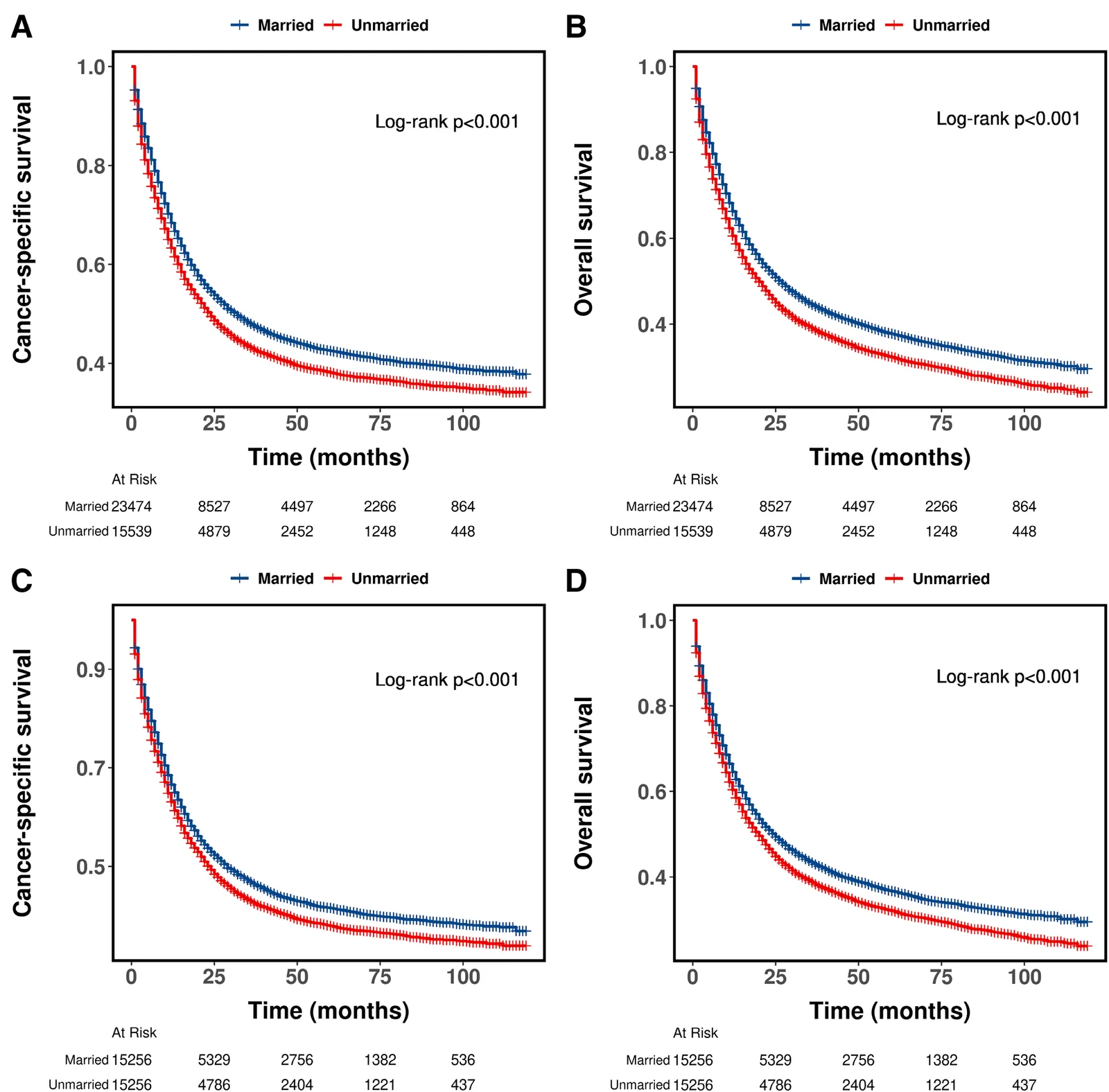
Kaplan–Meier curves for cancer-specific (A and C) and overall (B and D) survival before (A and B) and after (C and D) propensity-score matching.

During a median follow-up of 14 months, 19,008 of the 39,013 patients (48.7%) died of gastric cancer, and 21,470 (55.0%) died of any cause. Pre-PSM, unmarried patients had significantly worse cancer-specific survival (CSS: HR = 1.17, 95% confidence interval [CI] 1.14–1.20, *P* < .001; Fig. 2A) and overall survival (OS: HR = 1.20, 95% CI 1.16–1.23, *P* < .001; Fig. 2B) than married patients. Following 1:1 PSM, the survival gap narrowed but remained statistically significant (CSS: HR = 1.12, 95% CI 1.09–1.16, *P* < .001, Fig. 2C; OS: HR = 1.15, 95% CI 1.12–1.19, *P* < .001, Fig. 2D). Table 2 details CSS and OS at 12, 36, and 60 months. Before matching, unmarried patients had lower CSS at 12 (63.3% vs 68.4%), 36 (43.2% vs 48.0%), and 60 months (38.2% vs 42.5%), and lower OS at 12 (60.5% vs 66.3%), 36 (39.0% vs 44.5%), and 60 months (32.3% vs 37.7%); all differences were significant (*P* < .001). After matching, the pattern persisted: unmarried patients continued to show lower CSS at 12 (63.1% vs 66.6%), 36 (42.9% vs 46.8%), and 60 months (37.9% vs 41.5%), and lower OS at 12 (60.3% vs 64.5%), 36 (38.8% vs 43.4%), and 60 months (32.1% vs 36.7%); all *P* values <.001.

**Table 2:** Differences in cancer-specific survival and overall survival at 12, 36, and 60 months between marital status before and after propensity-score matching.

Characteristic	12-Month	36-Month	60-Month	*P* value*
CSS before PSM				
Overall	66.4% (65.9%, 66.9%)	46.1% (45.5%, 46.6%)	40.8% (40.2%, 41.4%)	
Married	68.4% (67.8%, 69.0%)	48.0% (47.2%, 48.7%)	42.5% (41.8%, 43.3%)	<.001
Unmarried	63.3% (62.5%, 64.1%)	43.2% (42.3%, 44.1%)	38.2% (37.2%, 39.1%)	
OS before PSM				
Overall	64.0% (63.5%, 64.5%)	42.3% (41.8%, 42.9%)	35.6% (35.0%, 36.2%)	
Married	66.3% (65.6%, 66.9%)	44.5% (43.8%, 45.2%)	37.7% (37.0%, 38.5%)	<.001
Unmarried	60.5% (59.7%, 61.3%)	39.0% (38.1%, 39.9%)	32.3% (31.5%, 33.3%)	
CSS after PSM				
Overall	64.8% (64.3%, 65.4%)	44.9% (44.2%, 45.5%)	39.7% (39.1%, 40.4%)	
Married	66.6% (65.8%, 67.3%)	46.8% (45.9%, 47.7%)	41.5% (40.6%, 42.5%)	<.001
Unmarried	63.1% (62.3%, 63.9%)	42.9% (42.0%, 43.8%)	37.9% (37.0%, 38.9%)	
OS after PSM				
Overall	62.4% (61.9%, 63.0%)	41.1% (40.4%, 41.7%)	34.4% (33.8%, 35.1%)	
Married	64.5% (63.7%, 65.3%)	43.4% (42.5%, 44.2%)	36.7% (35.8%, 37.6%)	<.001
Unmarried	60.3% (59.5%, 61.1%)	38.8% (37.9%, 39.6%)	32.1% (31.2%, 33.0%)	

CSS = cancer-specific survival, OS = overall survival, PSM = propensity score-matching.

* Log-rank test.

**Figure 3.**
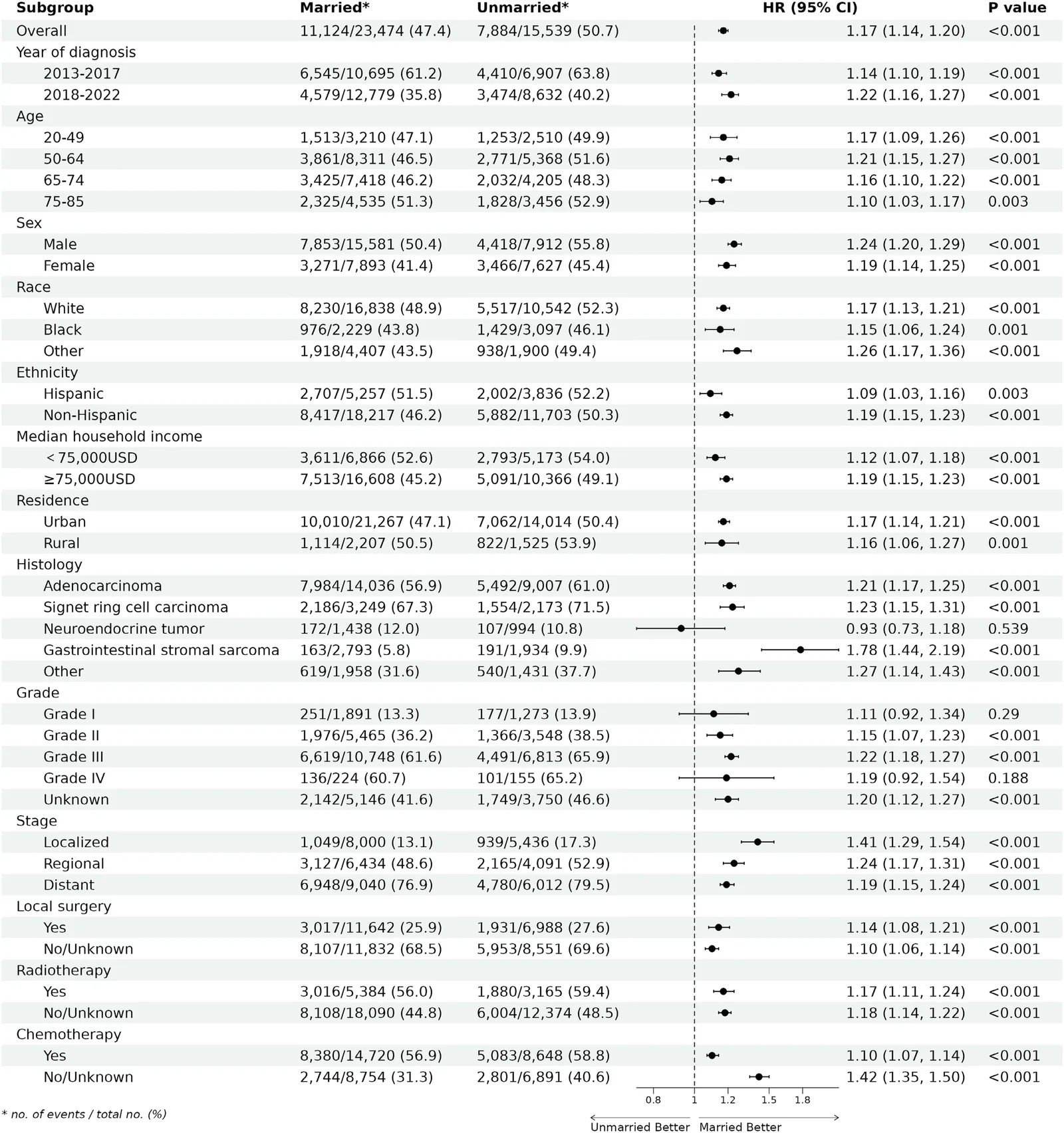
Forest plot of subgroup analysis of cancer-specific survival. CI = confidence interval, HR = hazard ratio.

**Figure 4.**
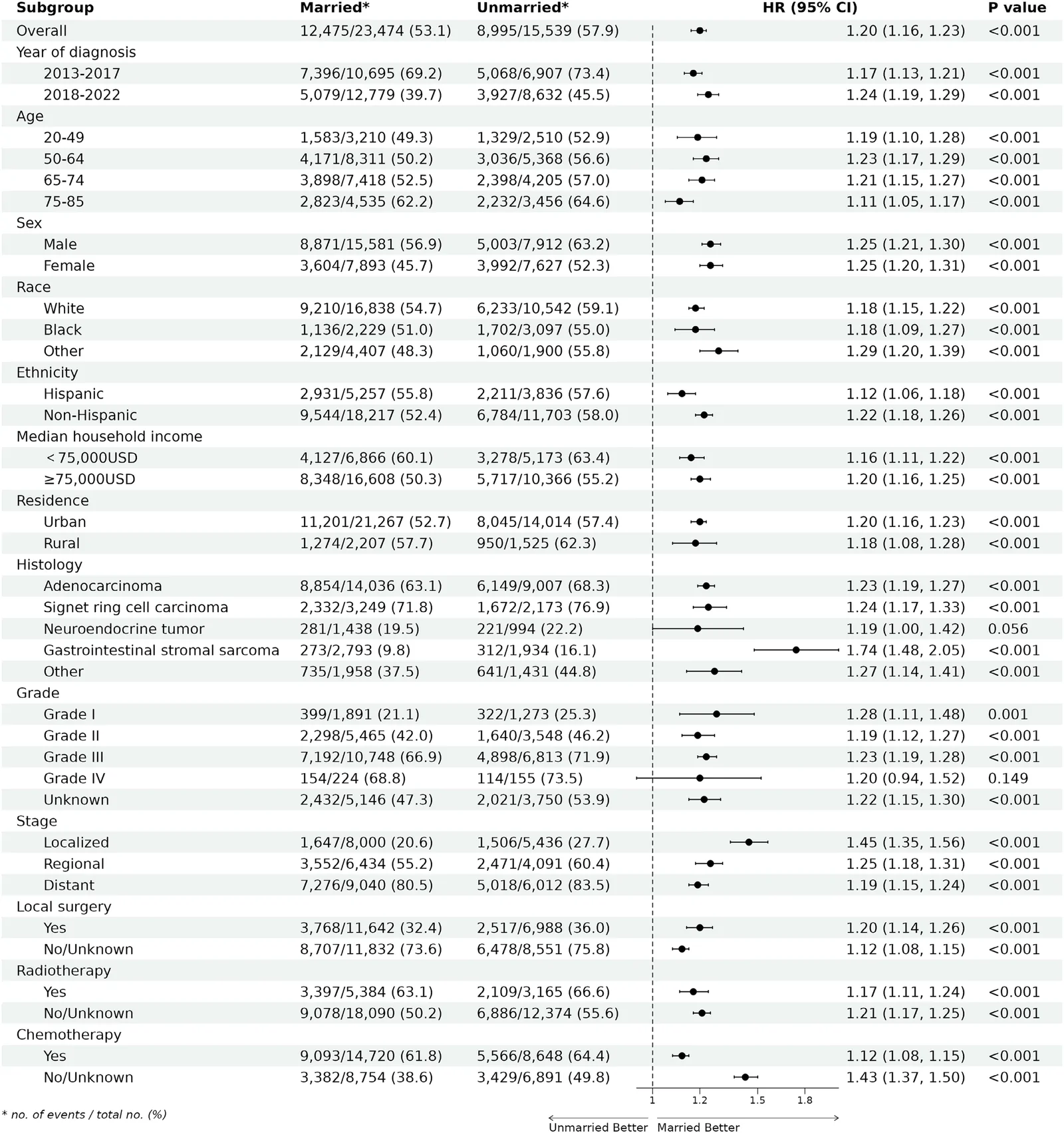
Forest plot of subgroup analysis of overall survival. CI = confidence interval, HR = hazard ratio.

The association between marital status and survival was then evaluated across 13 prespecified subgroups in the full cohort of 39,013 patients. Subgroup analyses are displayed in Figure 3 (CSS) and Figure 4 (OS). A significant marital-status effect on CSS was observed in virtually all subgroups except neuroendocrine histology and grade I or IV tumors (all *P* > .05). Likewise, OS was significantly lower among unmarried patients in all subgroups except neuroendocrine histology and grade IV tumors (all *P* > .05).

In multivariable Cox regression analyses of the entire cohort (n = 39,013), year of diagnosis, age, sex, race, marital status, median household income, residence, histologic type, tumor grade, stage, surgery, radiotherapy, and chemotherapy were identified as independent prognostic factors for both CSS and OS (all *P* < .05; Table 3). Ethnicity (Hispanic vs non-Hispanic) was not an independent predictor of either CSS or OS (*P* > .05). The *E* values for the multivariable associations between unmarried status and mortality were 1.62 (lower 95% confidence bound, 1.51) for CSS and 1.67 (lower 95% confidence bound, 1.57) for OS, indicating that an unmeasured confounder would need to be associated with both marital status and mortality by a risk ratio of at least 1.6 to 1.7, above and beyond all adjusted covariates, to fully explain away the observed associations.

**Table 3:** Univariate and multivariate Cox regression for cancer-specific survival and overall survival.

Characteristic	Cancer-specific survival				Overall survival			
	Univariate		Multivariate		Univariate		Multivariate	
	HR (95% CI)	*P* value	HR (95% CI)	*P* value	HR (95% CI)	*P* value	HR (95% CI)	*P* value
Year of diagnosis								
2013–2017	Ref		Ref		Ref		Ref	
2018–2022	0.75 (0.73–0.77)	<.001	0.84 (0.82–0.87)	<.001	0.77 (0.75–0.79)	<.001	0.86 (0.84–0.89)	<.001
Age								
20–49	Ref		Ref		Ref		Ref	
50–64	1.00 (0.95–1.04)	.862	1.04 (0.99–1.08)	.125	1.03 (0.98–1.07)	.235	1.06 (1.01–1.10)	.016
65–74	0.99 (0.95–1.04)	.782	1.12 (1.07–1.18)	<.001	1.09 (1.04–1.14)	<.001	1.21 (1.15–1.26)	<.001
75–85	1.22 (1.16, 1.27)	<.001	1.32 (1.26, 1.39)	<.001	1.41 (1.35, 1.48)	<.001	1.49 (1.42, 1.57)	<.001
Sex								
Male	Ref		Ref		Ref		Ref	
Female	0.78 (0.76–0.80)	<.001	0.92 (0.89–0.95)	<.001	0.77 (0.75–0.79)	<.001	0.89 (0.87–0.92)	<.001
Race								
White	Ref		Ref		Ref		Ref	
Black	0.88 (0.84–0.92)	<.001	1.05 (1.00–1.10)	.044	0.92 (0.88–0.96)	<.001	1.09 (1.04–1.13)	<.001
Other	0.84 (0.80–0.87)	<.001	0.90 (0.86–0.94)	<.001	0.83 (0.80–0.86)	<.001	0.88 (0.85–0.92)	<.001
Ethnicity								
Hispanic	Ref		Ref		Ref		Ref	
Non-Hispanic	0.85 (0.82–0.87)	<.001	0.97 (0.94–1.01)	.133	0.88 (0.85–0.91)	<.001	0.98 (0.95–1.01)	.258
Marital status								
Married	Ref		Ref		Ref		Ref	
Unmarried	1.17 (1.14–.20)	<.001	1.17 (1.13–1.20)	<.001	1.20 (1.16–1.23)	<.001	1.19 (1.15–1.22)	<.001
Median household income								
<75,000 USD	Ref		Ref		Ref		Ref	
≥75,000 USD	0.89 (0.87–0.92)	<.001	0.88 (0.85–0.91)	<.001	0.87 (0.85–0.89)	<.001	0.86 (0.84–0.89)	<.001
Residence								
Urban	Ref		Ref		Ref		Ref	
Rural	1.12 (1.07–1.17)	<.001	1.09 (1.03–1.15)	.002	1.14 (1.10–1.20)	<.001	1.09 (1.04–1.14)	<.001
Histology								
Adenocarcinoma	Ref		Ref		Ref		Ref	
Signet-ring cell carcinoma	1.28 (1.24–1.33)	<.001	1.19 (1.14–1.23)	<.001	1.24 (1.19–1.28)	<.001	1.19 (1.14–1.23)	<.001
Neuroendocrine tumor	0.13 (0.12–0.15)	<.001	0.28 (0.25–0.32)	<.001	0.21 (0.19–0.23)	<.001	0.37 (0.33–0.41)	<.001
Gastrointestinal stromal sarcoma	0.09 (0.08–0.10)	<.001	0.20 (0.18–0.22)	<.001	0.13 (0.12–0.15)	<.001	0.25 (0.23–0.28)	<.001
Other	0.56 (0.53–0.60)	<.001	0.49 (0.46–0.53)	<.001	0.60 (0.57–0.64)	<.001	0.51 (0.48–0.55)	<.001
Grade								
Grade Ⅰ	Ref		Ref		Ref		Ref	
Grade Ⅱ	3.60 (3.26–3.99)	<.001	1.50 (1.35–1.67)	<.001	2.59 (2.39–2.80)	<.001	1.28 (1.18–1.39)	<.001
Grade Ⅲ	7.65 (6.94–8.42)	<.001	2.21 (1.99–2.45)	<.001	5.11 (4.74–5.51)	<.001	1.84 (1.69–2.00)	<.001
Grade Ⅳ	5.57 (4.75–6.52)	<.001	3.13 (2.66–3.68)	<.001	3.74 (3.25–4.31)	<.001	2.46 (2.13–2.84)	<.001
Unknown	4.74 (4.29–5.24)	<.001	1.81 (1.63–2.01)	<.001	3.31 (3.06–3.59)	<.001	1.50 (1.38–1.64)	<.001
Stage								
Localized	Ref		Ref		Ref		Ref	
Regional	4.06 (3.85–4.27)	<.001	3.62 (3.42–3.83)	<.001	2.96 (2.84–3.09)	<.001	2.77 (2.64–2.91)	<.001
Distant	11.78 (11.23–12.37)	<.001	6.47 (6.12–6.85)	<.001	8.11 (7.79–8.44)	<.001	4.84 (4.61–5.08)	<.001
Local surgery								
Yes	Ref		Ref		Ref		Ref	
No/unknown	5.18 (5.01–5.36)	<.001	3.23 (3.10–3.37)	<.001	4.60 (4.46–4.74)	<.001	3.05 (2.94–3.16)	<.001
Radiotherapy								
Yes	Ref		Ref		Ref		Ref	
No/unknown	0.85 (0.82–0.87)	<.001	0.92 (0.89–0.95)	<.001	0.85 (0.82–0.87)	<.001	0.92 (0.89–0.95)	<.001
Chemotherapy								
Yes	Ref		Ref		Ref		Ref	
No/unknown	0.60 (0.58–0.62)	<.001	2.12 (2.05–2.20)	<.001	0.66 (0.65–0.68)	<.001	2.07 (2.00–2.14)	<.001

CI = confidence interval, HR = hazard ratio.

### 3.3. Sensitivity analysis of marital-status subgroups

Using the original 6-category marital-status variable, 7505 patients (19.2%) were single (never married), 529 (1.4%) separated, 3635 (9.3%) divorced, 3594 (9.2%) widowed, and 276 (0.7%) unmarried or living with a domestic partner (Table S1, Supplemental Digital Content 1). Median CSS ranged from 21 months in separated patients to 32 months in married patients, and median OS from 18 to 27 months (overall log-rank *P* < .001 for both CSS and OS; Table S2, Supplemental Digital Content 2). In multivariable Cox regression with married patients as the reference, single (CSS: HR 1.20, 95% CI 1.15–1.24; OS: HR 1.21, 95% CI 1.16–1.25), divorced (CSS: HR 1.17, 95% CI 1.12–1.23; OS: HR 1.21, 95% CI 1.15–1.26), and widowed patients (CSS: HR 1.13, 95% CI 1.07–1.19; OS: HR 1.16, 95% CI 1.10–1.21) all had significantly worse CSS and OS after full adjustment (all *P* < .001). Separated patients showed a nonsignificant trend toward worse CSS (HR 1.09, 95% CI 0.97–1.23, *P* = .148) and significantly worse OS (HR 1.13, 95% CI 1.01–1.26, *P* = .032); the small size of this subgroup (n = 529) limited statistical power. Notably, patients who were unmarried but living with a domestic partner had outcomes statistically indistinguishable from married patients (CSS: HR 0.99, 95% CI 0.83–1.18; OS: HR 0.97, 95% CI 0.82–1.15), suggesting that the presence of a supportive partner, rather than the legal formality of marriage, underlies most of the observed benefit (Tables S1 and S2, Supplemental Digital Content 1).

## 4. Discussion

Using a large, retrospective cohort extracted from the SEER database, we found that unmarried patients with gastric cancer experienced significantly worse outcomes than their married counterparts, and that marital status represents an independent prognostic factor. After baseline imbalances were adjusted with PSM and covariates were controlled in multivariable Cox models, the survival disadvantage persisted. Notably, except for the small subsets with neuroendocrine histology or grade I/IV tumors, unmarried patients had consistently poorer CSS across all subgroups (HR > 1, *P* < .05); similarly, OS was inferior in every subgroup except neuroendocrine histology and grade IV tumors (*P* > .05). The lack of statistical significance in grade IV disease probably reflects limited power due to small sample size, and the same explanation may apply to grade I tumors.

Marriage has repeatedly been linked to cancer prognosis.^[4]^ A landmark analysis by Aizer et al covering the 10 leading U.S. cancer sites showed that married patients were less likely to present with metastatic disease (odds ratio = 0.83, 95% CI 0.82–0.84), more likely to receive definitive therapy (odds ratio = 1.53, 95% CI 1.51–1.56), and less likely to die of their cancer (HR = 0.80, 95% CI 0.79–0.81), with the benefit persisting in site-specific models (all *P* < .001).^[5]^ Comparable findings have been reported for non-small-cell lung cancer,^[13]^ breast cancer,^[14]^ prostate cancer,^[15,16]^ glioblastoma,^[17]^ early hepatocellular carcinoma,^[18]^ pancreatic ductal adenocarcinoma,^[19]^ and gynecologic malignancies.^[20–25]^ Previous gastric-cancer studies were small, used pre-2012 data,^[7–9]^ or focused on single histologic subtypes,^[10,11]^ leaving their conclusions vulnerable to temporal and selection bias. As living standards and treatment paradigms evolve, the biopsychosocial relevance of marriage may also change. Our study – 39,013 patients, all histologic types, multi-ethnic, 2013 to 2022 – provides the largest, most contemporary update. The survival benefit associated with marriage remained intact after modern staging and therapy, indicating that marital status is a quantifiable and potentially modifiable prognostic factor, not merely a descriptive association.

Our findings are broadly consistent with and extend the existing evidence. A systematic review and meta-analysis encompassing >6 million patients with cancer concluded that unmarried status confers a significantly higher risk of cancer-specific and overall mortality,^[4]^ and the landmark analysis by Aizer et al of the 10 leading U.S. cancer sites demonstrated substantially lower cancer-specific mortality among married patients.^[5]^ For gastric cancer specifically, our adjusted estimates (CSS: HR 1.17; OS: HR 1.19) are remarkably consistent with earlier SEER-based studies: Jin et al and Zhou et al reported that unmarried patients with gastric cancer had approximately 15% to 25% higher mortality^[7,8]^; Li and Hu confirmed a persistent, time-varying protective effect of marriage^[9]^; and studies restricted to gastric adenocarcinoma reached similar conclusions.^[10]^ The concordance between our contemporary estimates and these earlier observations, despite substantial changes in staging practice and systemic therapy over the past decade, indicates that the prognostic value of marital status is robust to temporal changes in treatment paradigms.

Nevertheless, our findings diverge from previous reports in some respects. Zhou et al identified marital status as an independent prognostic factor in gastric neuroendocrine neoplasms,^[11]^ whereas in our substantially larger neuroendocrine subgroup (n = 2432), the association between marital status and CSS did not reach statistical significance. This discrepancy may reflect differences in sample size, era of diagnosis, and covariate adjustment. In addition, the neuroendocrine subgroup in our cohort had a markedly lower baseline cancer-specific mortality than adenocarcinoma (approximately 11.5% vs 58.5%; Fig. 3), consistent with a predominance of well-differentiated neuroendocrine tumors; such low event rates reduce the statistical power to detect modest survival differences related to social determinants. Similarly, no significant association was observed for grade I or grade IV tumors, which likely reflects limited statistical power in these small subgroups. These discrepancies indicate that the prognostic impact of marital status is not uniform across all biological contexts and should not be assumed to be universal.

The present study adds several novel elements to the existing evidence. First, based on 39,013 eligible patients identified from the SEER database among those diagnosed with gastric cancer during 2013 to 2022, this is, to our knowledge, the largest and most contemporary analysis of marital status and gastric-cancer prognosis, reflecting modern staging and treatment paradigms. Second, the combination of propensity-score matching and multivariable Cox regression, together with consistent effect estimates across nearly all prespecified subgroups, strengthens the credibility of the association. Third, our disaggregated sensitivity analysis of unmarried subgroups provides novel evidence that the survival disadvantage is shared by single, divorced, and widowed patients, whereas patients living with a domestic partner had outcomes comparable to married patients (Table S1, Supplemental Digital Content 1) – supporting the interpretation that the benefit of “marriage” is driven largely by the presence of a supportive partner rather than by legal marital status per se. Finally, we quantified the robustness of our findings to unmeasured confounding using *E* values.

Disaggregating the unmarried group confirmed that heterogeneity exists within this category. Single, divorced, and widowed patients each had significantly worse CSS and OS than married patients after full adjustment, with adjusted HRs ranging from 1.13 to 1.21 (Table S1, Supplemental Digital Content 1). Widowed patients had the largest unadjusted OS hazard (HR 1.29), which attenuated to 1.16 after adjustment, indicating that their excess mortality is partly explained by older age and other baseline differences rather than widowhood per se. The separated subgroup (n = 529) showed a similar direction of effect that reached significance for OS but not CSS, likely reflecting limited statistical power. Most notably, patients living with a domestic partner had outcomes statistically indistinguishable from married patients, supporting the interpretation that the availability of a supportive partner, rather than the legal formality of marriage, drives most of the observed survival benefit.

Current evidence suggests that the effect of marital status on malignant disease outcomes may operate through several pathways. Biologically, studies indicate that marriage can improve physiologic status by influencing cardiovascular, endocrine, and immune function, with marital quality playing a decisive role.^[26,27]^ A loving, supportive partner is associated with increased oxytocin release, which can inhibit tumor growth through direct and indirect mechanisms.^[28]^ In contrast, unmarried patients may be chronically exposed to higher levels of glucocorticoids and catecholamines, leading to physiologic dysfunction, negative remodeling of the tumor microenvironment, enhanced tumor growth and migration, and stimulated angiogenesis, all of which can worsen prognosis.^[29]^ Psychologically, stress and depression reduce cytotoxic T-cell and natural-killer-cell activity, weakening immunosurveillance and promoting tumor progression.^[30,31]^ Widowed individuals have been shown to exhibit poorer peripheral-blood lymphocyte responses and lower natural-killer-cell activity – cells critical for recognizing and eliminating cancer cells.^[4]^ Socially and psychologically, cancer patients experience substantial psychological stress; emotional support provided by a spouse can mitigate the negative effects of stress and improve treatment efficacy.^[14]^ A healthy marriage helps maintain a positive mood, reduces anxiety and depression, and is essential for improving survival.^[32,33]^ Widowed, divorced, or separated individuals are more vulnerable to psychological distress and mental-health problems because they lack partner support during diagnosis and treatment.^[34]^ With emotional encouragement from a partner, married patients are better able to cope with stress and depression and are more motivated to seek treatment.^[35,36]^ Prospective studies have confirmed that optimism is associated with lower mortality risk.^[37]^ Regarding adherence, married individuals usually adopt healthier lifestyles – including better diet, more exercise, and less substance abuse – and stable marriages improve treatment adherence; patients from cohesive families display higher adherence than those from conflict-ridden families, and higher adherence is closely linked to better survival.^[38–41]^ Economically, stable marriage is usually associated with higher socioeconomic status, and family members such as spouses and children may provide long-term financial and emotional support for treatment.^[42]^ Married individuals with better economic status are more likely to purchase health insurance and obtain medical subsidies at diagnosis.^[43]^ Additionally, married patients are more likely to be diagnosed at an early stage and to choose active treatment; they are more likely to receive chemotherapy and to enjoy higher survival rates than unmarried patients, possibly because of family involvement in treatment planning and a higher perceived value of life.^[15]^

Finally, the observed survival benefit should be interpreted pragmatically: in the U.S. healthcare context, marriage is to a large extent a surrogate marker of socioeconomic resilience. Married patients are more likely to have dual household incomes, more comprehensive health-insurance coverage, and a dedicated caregiver who provides transportation to treatment sessions, manages complex medication schedules, and facilitates timely follow-up. A mediation analysis in a SEER-based cohort estimated that insurance status mediates a considerable proportion of the association between marital status and cancer-specific survival,^[43]^ and socioeconomic disadvantage is a well-established determinant of adverse cancer outcomes.^[42]^ Consistent with this interpretation, the association in our cohort persisted but attenuated after adjustment for county-level median household income and urban/rural residence, and patients living with a domestic partner – who share the tangible support structure of marriage without its legal formality – had outcomes statistically indistinguishable from married patients (Table S1, Supplemental Digital Content 1). Taken together, these observations indicate that tangible socioeconomic and logistical resources, rather than any intrinsic biological effect of marriage per se, likely drive most of the observed benefit. The biological hypotheses discussed above (e.g., neuroendocrine-immune modulation) should therefore be regarded as speculative and secondary to these tangible socioeconomic and logistical mechanisms.

Our study has several strengths. First, by leveraging one of the largest cancer databases worldwide – the SEER registry – we had access to a huge patient resource, which enhanced statistical power and broad representativeness. Second, by applying advanced statistical methods such as PSM and multivariable Cox regression, we minimized the influence of baseline imbalances and covariates, thereby increasing the credibility of our findings. Third, compared with previous reports, our study incorporated a much larger, more recent sample and controlled for more potential confounders, markedly improving precision. Nevertheless, limitations exist. First, as a retrospective analysis, our study is more susceptible to bias than prospective investigations. Second, SEER does not capture several important prognostic variables – most notably Eastern Cooperative Oncology Group performance status, lifestyle factors such as tobacco and alcohol use, validated comorbidity indices such as the Charlson comorbidity score, detailed chemotherapy regimens and doses, and specific pathologic features. Because healthier and fitter individuals are more likely to marry and to remain married, and are also better able to tolerate gastric-cancer treatment, part of the observed survival difference may reflect differences in baseline fitness and lifestyle rather than marital support itself; such healthy-selection confounding would generally be expected to bias the observed association away from the null. Although propensity-score matching balanced all measured covariates, it cannot balance unmeasured confounders. Our *E* value analysis indicates that an unmeasured confounder would need to be associated with both marital status and mortality by a risk ratio of at least 1.6 to 1.7, above and beyond all adjusted covariates, to fully explain away the observed associations; nevertheless, residual confounding cannot be excluded, and prospective studies incorporating performance status, lifestyle factors, and validated comorbidity indices are warranted. Third, SEER captures marital status at the time of diagnosis only and does not record subsequent transitions – divorce, separation, or widowhood – that may occur during the physically and emotionally demanding course of cancer treatment; nor does it capture new partnerships formed after diagnosis. Such transitions would move patients between exposure groups over time, and the resulting misclassification is likely non-differential with respect to outcome, which would be expected to bias the observed association toward the null; the true protective effect of sustained spousal support may therefore be stronger than our estimates suggest. Prospective cohorts with repeated assessment of marital status are needed to capture the dynamic nature of social support during cancer treatment. Moreover, SEER does not record marital quality, limiting our ability to explore how relationship quality affects gastric-cancer outcomes. Studies show that relationship closeness importantly influences psychological adjustment^[44]^; poor marital quality can produce greater functional impairment and worse health behaviors, ultimately worsening prognosis.^[45]^ Fourth, SEER documents only legally recognized marriages; de-facto cohabiting partnerships are classified as unmarried. Although the proportion of such patients is small, they may receive emotional and socioeconomic support equivalent to that of married patients, potentially biasing our results. Furthermore, although our dichotomization of marital status follows the conventions of the literature, it pools single, separated, divorced, and widowed patients, whose psychosocial and financial circumstances differ; our disaggregated sensitivity analysis (Table S1, Supplemental Digital Content 1) suggests broadly comparable survival disadvantages for single, divorced, and widowed patients, but statistical power was limited in the smaller subgroups, and residual heterogeneity within the unmarried category cannot be excluded. Finally, because SEER is composed largely of White and Black populations, generalizability to other racial groups – Asians, for example – may be limited. Well-designed, large-scale prospective studies that include more racial and ethnic groups are therefore needed to confirm the impact of marital status on gastric-cancer prognosis.

## 5. Conclusion

In summary, our large-sample analysis demonstrates that unmarried status is significantly associated with worse gastric-cancer outcomes and confirms marital status as an independent prognostic indicator. To validate these findings and to elucidate the underlying biologic mechanisms, well-powered prospective studies and mechanistic investigations are urgently needed. Additional research should also examine whether marital satisfaction and relationship quality influence gastric-cancer prognosis.

## Author contributions

**Conceptualization:** Haitao Ma, Yongyong Liu.

**Data curation:** Haitao Ma, Qiang Bing, Xuhao Duan, Genwu Yao, Bichen Wang, Yaozu Bao.

**Formal analysis:** Haitao Ma, Bichen Wang.

**Funding acquisition:** Haitao Ma, Yongyong Liu.

**Investigation:** Qiang Bing.

**Methodology:** Haitao Ma, Yaozu Bao.

**Project administration:** Yongyong Liu.

**Resources:** Yongyong Liu.

**Software:** Haitao Ma, Xuhao Duan, Genwu Yao.

**Supervision:** Yongyong Liu.

**Validation:** Haitao Ma, Qiang Bing.

**Visualization:** Xuhao Duan.

**Writing – original draft:** Haitao Ma, Qiang Bing, Xuhao Duan, Genwu Yao, Bichen Wang, Yaozu Bao, Yongyong Liu.

**Writing – review & editing:** Haitao Ma, Qiang Bing, Xuhao Duan, Genwu Yao, Bichen Wang, Yaozu Bao, Yongyong Liu.




