# Supplementary material for: The impact of marital status on gastric cancer-related prognosis: A propensity-score matched study
Source: Medicine (Baltimore). 2026 Sep 18;105(38):e50761. doi: 10.1097/MD.0000000000050761 (PMC13593193; doi:10.1097/MD.0000000000050761)
Supplement: Supplementary file 1 [file medi-105-e50761-s001.docx]

**Supplementary Table S1. Sensitivity analysis of marital-status subgroups: distribution and Cox regression for cancer-specific survival (CSS) and overall survival (OS) in the full cohort (n = 39,013).**

| **Marital status** | **N (%)** | **CSS univariate HR (95% CI), p** | **CSS multivariate HR (95% CI), p*** | **OS univariate HR (95% CI), p** | **OS multivariate HR (95% CI), p*** |
| --- | --- | --- | --- | --- | --- |
| Married (reference) | 23,474 (60.2) | Reference | Reference | Reference | Reference |
| Single (never married) | 7,505 (19.2) | 1.18 (1.14–1.23), <0.001 | 1.20 (1.15–1.24), <0.001 | 1.18 (1.14–1.22), <0.001 | 1.21 (1.16–1.25), <0.001 |
| Separated | 529 (1.4) | 1.21 (1.08–1.36), 0.001 | 1.09 (0.97–1.23), 0.148 | 1.23 (1.10–1.37), <0.001 | 1.13 (1.01–1.26), 0.032 |
| Divorced | 3,635 (9.3) | 1.12 (1.07–1.18), <0.001 | 1.17 (1.12–1.23), <0.001 | 1.15 (1.10–1.21), <0.001 | 1.21 (1.15–1.26), <0.001 |
| Widowed | 3,594 (9.2) | 1.20 (1.14–1.26), <0.001 | 1.13 (1.07–1.19), <0.001 | 1.29 (1.23–1.35), <0.001 | 1.16 (1.10–1.21), <0.001 |
| Unmarried or domestic partner | 276 (0.7) | 1.04 (0.87–1.24), 0.641 | 0.99 (0.83–1.18), 0.925 | 1.01 (0.85–1.19), 0.926 | 0.97 (0.82–1.15), 0.763 |

*Adjusted for year of diagnosis, age, sex, race, ethnicity, median household income, residence, histologic type, grade, stage, surgery, radiotherapy, and chemotherapy (same covariates as Table 3). HR, hazard ratio; CI, confidence interval.
