# Supplementary material for: The impact of marital status on gastric cancer-related prognosis: A propensity-score matched study
Source: Medicine (Baltimore). 2026 Sep 18;105(38):e50761. doi: 10.1097/MD.0000000000050761 (PMC13593193; doi:10.1097/MD.0000000000050761)
Supplement: Supplementary file 2 [file medi-105-e50761-s002.docx]

**Supplementary Table S2. Kaplan–Meier estimates of cancer-specific survival (CSS) and overall survival (OS) by marital-status subgroup in the full cohort (n = 39,013).**

| **Marital status** | **N (%)** | **Median CSS (months)** | **12-month CSS** | **36-month CSS** | **60-month CSS** | **Median OS (months)** | **12-month OS** | **36-month OS** | **60-month OS** |
| --- | --- | --- | --- | --- | --- | --- | --- | --- | --- |
| Married | 23,474 (60.2) | 32 | 68.4% | 48.0% | 42.5% | 27 | 66.3% | 44.5% | 37.7% |
| Single (never married) | 7,505 (19.2) | 23 | 62.9% | 42.6% | 37.6% | 20 | 60.5% | 39.1% | 33.0% |
| Separated | 529 (1.4) | 21 | 61.9% | 41.9% | 35.9% | 18 | 59.7% | 38.4% | 30.0% |
| Divorced | 3,635 (9.3) | 25 | 64.8% | 44.5% | 39.5% | 22 | 61.9% | 40.2% | 33.4% |
| Widowed | 3,594 (9.2) | 24 | 62.5% | 43.1% | 38.0% | 19 | 59.0% | 37.5% | 30.1% |
| Unmarried or domestic partner | 276 (0.7) | 25 | 68.2% | 43.5% | 42.5% | 23 | 67.2% | 40.7% | 38.7% |

Overall log-rank p < 0.001 for both CSS and OS across the six subgroups. CSS, cancer-specific survival; OS, overall survival.
